# Daytime Bladder Control Status in Toddlerhood Is Associated With Subsequent Bedwetting in Preschool Years: A Nationwide Cohort Study of Over 30 000 Japanese Children

**DOI:** 10.1111/iju.70288

**Published:** 2025-11-17

**Authors:** Takatoshi Moriwake, Naomi Matsumoto, Yusuke Tominaga, Kensuke Uraguchi, Tomoko Kobayashi, Ichiro Tsuboi, Kasumi Yoshinaga, Tomoaki Yamanoi, Tatsushi Kawada, Takuya Sadahira, Satoshi Katayama, Takehiro Iwata, Shingo Nishimura, Kensuke Bekku, Kohei Edamura, Soshi Takao, Takashi Yorifuji, Motoo Araki

**Affiliations:** ^1^ Department of Urology Graduate School of Medicine, Dentistry and Pharmaceutical Sciences, Okayama University Okayama Japan; ^2^ Department of Epidemiology Graduate School of Medicine, Dentistry and Pharmaceutical Sciences, Okayama University Okayama Japan

**Keywords:** bedwetting, cohort study, daytime bladder control, nocturnal enuresis

## Abstract

**Objectives:**

Nocturnal enuresis is common in early childhood. While daytime bladder control typically precedes nighttime continence, the temporal relationship between early daytime bladder control and subsequent bedwetting remains unclear. We investigated whether daytime bladder control status at age 2.5 years—as indicated by diaper use—is associated with bedwetting at age 4.5 years in a Japanese nationwide cohort.

**Methods:**

We analyzed data from the Japanese Longitudinal Survey of Newborns in the 21st Century (2010 cohort). Daytime bladder control was assessed at age 2.5 years through caregiver‐reported diaper use, and bedwetting frequency at age 4.5 years through parental questionnaires. Modified Poisson regression estimated risk ratios (RRs), adjusting for birth‐related factors, socioeconomic status, daycare attendance, and developmental milestones.

**Results:**

Among 32 168 children, 26 651 (82.8%) still used diapers at 2.5 years. Bedwetting prevalence at 4.5 years was 42.2%: 34.5% in children who achieved daytime bladder control at 2.5 years versus 43.9% in those still using diapers. After multivariable adjustment, incomplete daytime bladder control at 2.5 years was associated with higher bedwetting risk (adjusted RR 1.25; 95% CI, 1.20–1.31). Multinomial regression revealed dose–response relationships: odds ratios 1.41 (95% CI, 1.30–1.52) for “sometimes” and 1.58 (95% CI, 1.42–1.77) for “often” bedwetting.

**Conclusions:**

Daytime bladder control status at 2.5 years was associated with a 25% increased bedwetting risk at 4.5 years. This association likely reflects individual differences in bladder control maturation rather than causal effects. While daytime bladder control may serve as a developmental marker, its validity as an intervention target remains unestablished.

## 1 Introduction

Nocturnal enuresis (NE) is a common childhood condition that affects approximately 15% of 5‐year‐old children [1]. Although NE often resolves spontaneously as children mature, it can lead to low self‐esteem and social isolation [2, 3]. Additionally, NE impacts school performance, peer relationships, and family burden [4]. The delayed resolution of NE has also been linked to nocturnal awakenings and urinary urgency in adulthood [5], highlighting the need for understanding its developmental origins.

The development of bladder control typically follows a sequential pattern, with most children achieving daytime continence before nighttime dryness [6]. This involves maturation of multiple physiological systems including bladder capacity [7], cortical pathways [8], and hormonal rhythms [6]. Understanding the relationship between early daytime bladder control achievement and subsequent nocturnal continence may provide insights into identifying children at risk for persistent bedwetting.

Previous studies have identified various risk factors for NE, including genetic predisposition, constipation, developmental delays, and psychosocial factors [6, 9]. Additionally, associations between daytime bladder control and subsequent NE have been reported [10], and delayed achievement of bladder control has been linked to increased NE risk [11]. However, most existing studies are cross‐sectional in design with small sample sizes, limiting adequate adjustment for confounding factors. Furthermore, most research focused on children ≥ 5 years, leaving early development understudied.

To address this knowledge gap, we conducted a large‐scale longitudinal study using data from the Japanese Longitudinal Study of Children Born in the 21st Century, involving over 30 000 children. This is the first large‐scale study to examine daytime bladder control status in toddlerhood as a developmental marker rather than as an intervention effect of toilet training practices, and to investigate its association with subsequent bedwetting risk. Assessment of diaper‐free status at 2.5 years, preceding bedwetting evaluation at 4.5 years, establishes temporal sequence, reducing concerns about reverse causality. Comprehensive adjustments for confounding factors strengthen the validity of the findings.

The primary objective was to examine the association between daytime bladder control status at age 2.5 years—as indicated by continued diaper use—and bedwetting occurrence at age 4.5 years. The period from 2.5 to 4.5 years represents a critical developmental window during which children typically achieve daytime continence [7], and assessment at 4.5 years captures developmental trajectories before formal NE diagnosis (at age 5 or older). The availability of survey data at these time points also enabled this assessment. We hypothesized that children who have not achieved daytime continence by 2.5 years would show higher rates of bedwetting at 4.5 years, likely reflecting a developmental trajectory of bladder control maturation rather than necessarily a direct causal effect of diaper use per se. Understanding this developmental relationship may help identify children at risk for persistent bedwetting and inform future research on optimal support strategies.

## 2 Materials and Methods

### 2.1 Participants

One of the national‐scale birth cohort studies conducted in Japan is “The Longitudinal Survey of Newborns in the 21st Century”, performed by the Ministry of Health, Labour and Welfare. This study, which began with cohorts in 2001 and 2010.

The 2010 cohort included all children born nationwide between May 10 and May 24, 2010, including twins/triplets. Participants were identified from birth certificates. Annual mail surveys were conducted.

**FIGURE 1:**
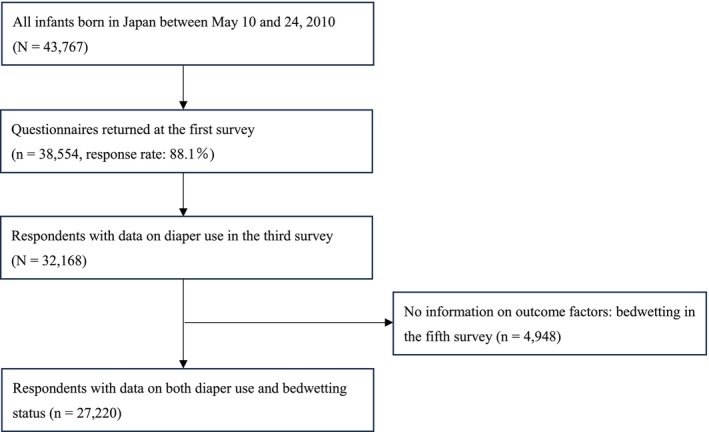
Flowchart for the selection of participants.

The response rate for the first survey was 88.1% (38 554/43 767). For our analysis, we excluded 6386 participants without information on diaper use, resulting in a baseline sample size of 32 168 children (Figure 1).

### 2.2 Ethical Considerations

This study adhered to the Declaration of Helsinki and Japanese Ethical Guidelines for Clinical Research. Ethical approval was obtained from the Institutional Review Board of Okayama University Hospital (approval number: 1506‐073). As this was a secondary analysis of de‐identified data, additional informed consent was waived.

### 2.3 Daytime Bladder Control Status (Exposure)

Daytime bladder control status was assessed using data from the third survey conducted at 2.5 years of age. Caregivers were asked, “Has your child stopped using diapers during the day?” with response options of “Yes” or “No.” This measure reflects bladder control development rather than training practices. Based on these responses, participants were classified into two groups: “Diaper‐Free” (children who no longer used diapers during the day) and “Diaper‐Using” (children who continued to use diapers during the day).

### 2.4 Bedwetting (Outcome Measures)

The primary outcome, bedwetting, was evaluated using data from the fifth survey conducted when children were 4.5 years of age. Caregivers were asked, “Does your child wet the bed?”, with three response options: “Never”, “Sometimes”, or “Often”. Responses were dichotomized: children whose caregivers answered “Never” were classified as the non‐bedwetting group, while those who answered “Sometimes” or “Often” were classified as the bedwetting group.

The term “bedwetting” was used in this study report instead of “nocturnal enuresis,” as NE is strictly defined as a condition in children aged 5 years or older.

### 2.5 Other Covariates

To account for potential confounding factors, we included the characteristics of both the child and the parent in our analysis.

### 2.6 Model 1: Birth‐Related and Socioeconomic Factors

Birth‐related variables included sex (male or female), birth order (first or second and later), birth weight (< 2500 g or ≥ 2500 g), and preterm birth (< 37 weeks or ≥ 37 weeks of gestation). Parental characteristics included age at the time of birth, paternal education and maternal education (categorized as ≤ junior high school, high school, junior college/vocational school, or 4‐year college or higher), and region of residence at birth (categorized as ward/district, city, town, or village). These variables were adjusted to account for perinatal and socioeconomic effects.

### 2.7 Model 2: Additional Adjustment for Postnatal Environmental Factors

In addition to all variables in Model 1, postnatal environmental factors were further adjusted in Model 2. These included daycare attendance and developmental milestones at 2.5 years of age, which, based on previous studies, may affect the outcome [12, 13, 14]. Daycare attendance was defined as childcare primarily provided by a daycare facility or babysitter on weekdays when the child was 2.5 years of age. Developmental milestones were based on CDC guidelines [15] for 30‐month‐old children.

The developmental indicators assessed in this study included:

- Gross motor skills: “Cannot climb stairs independently.”
- Language development: “Cannot speak in two‐word sentences.”
- Social habits/lifestyle skills: “Cannot dress or undress independently.”

Information on sex, birth order, birth weight, preterm birth, parental ages, and residence at birth was obtained from birth records. The educational levels of parents were derived from the second survey, while data on daycare attendance and developmental indicators were collected from the third survey.

### 2.8 Statistical Analysis

Participants were classified into “Diaper‐Free” and “Diaper‐Using” groups based on daytime bladder control status at 2.5 years of age. Selection bias was assessed by comparing demographic characteristics between included and lost‐to‐follow‐up children. Chi‐squared and t‐tests were used for categorical and continuous variables, respectively.

Modified Poisson regression with robust error variance was used to assess the association between daytime bladder control status and bedwetting. Crude risk ratios (RRs) with 95% confidence intervals (CIs) were first estimated (crude model). Adjusted analyzes were then performed in two steps: Model 1 included adjustments for birth‐related factors, parental age, education level, and residential area; Model 2 was further adjusted for daycare attendance and developmental indicators from the third survey, in addition to the factors in Model 1.

Complete case analysis was employed. Sensitivity analyzes using multinomial logistic regression examined bedwetting frequency as a three‐category outcome (Never/Sometimes/Often). For all statistical tests, a two‐sided *p*‐value of less than 0.05 was considered significant. Statistical analyzes were conducted using Stata version 17 (StataCorp LP, College Station, TX, USA).

## 3 Results

### 3.1 Demographic Characteristics

After excluding 6386 participants without information on diaper use, the baseline sample comprised 32 168 children (16 562 boys and 15 606 girls) (Figure 1). The demographic characteristics of the study participants, stratified by bladder control status at 2.5 years of age, are presented in Table 1. Among the 32 168 eligible children, 5517 (17.2%) were diaper‐free, while 26 651 (82.8%) were using diapers.

**TABLE 1:** Demographic characteristics of eligible children by daytime bladder control status at 2.5 years of age.

	Total (*N* = 32 168)	Diaper‐Free (*n* = 5517)	Diaper‐Using (*n* = 26 651)
Birth information			
Sex, *n* (%)			
Boys	16 562 (51.5)	2435 (44.1)	14 127 (53.0)
Girls	15 606 (48.5)	3082 (55.9)	12 524 (47.0)
Birth order, *n* (%)^a^			
First	15 076 (46.9)	2323 (42.1)	12 753 (47.9)
Second or later	17 092 (53.1)	3194 (57.9)	13 898 (52.1)
Birth weight, *n* (%)^a^			
< 2500 g	2983 (9.3)	393 (7.1)	2590 (9.7)
≥ 2500 g	29 185 (90.7)	5124 (92.9)	24 061 (90.3)
Gestational age, *n* (%)^a^			
Term (≥ 37 weeks)	30 492 (94.8)	5319 (96.4)	25 173 (94.5)
Preterm (< 37 weeks)	1676 (5.2)	198 (3.6)	1478 (5.5)
Behavioral development and other factors at 2.5 years of age			
Daycare attendance, *n* (%)^c^			
No	20 550 (63.9)	2761 (50.1)	17 789 (66.8)
Yes	11 604 (36.1)	2753 (49.9)	8851 (33.2)
Missing	14 (0.04)	3 (0.05)	11 (0.04)
Gross motor skills, *n* (%)^c^			
Unable to walk alone	115 (0.4)	2 (0.04)	113 (0.4)
Missing	6 (0.02)	1 (0.02)	5 (0.02)
Unable to run	210 (0.7)	2 (0.04)	208 (0.8)
Missing	5 (0.02)	0 (0)	5 (0.02)
Unable to climb stairs without help	654 (2.0)	40 (0.7)	614 (2.3)
Missing	12 (0.04)	1 (0.02)	11 (0.04)
Language development, *n* (%)^c^			
Unable to say meaningful words	241 (0.8)	4 (0.07)	237 (0.9)
Missing	5 (0.02)	2 (0.04)	3 (0.01)
Unable to speak in two‐word phrases	1395 (4.3)	43 (0.8)	1352 (5.1)
Missing	11 (0.03)	1 (0.02)	10 (0.04)
Unable to say own name	3667 (11.4)	184 (3.3)	3483 (13.1)
Missing	54 (0.17)	7 (0.13)	47 (0.18)
Social skills and lifestyle habits, *n* (%)^c^			
Unable to eat without help using a spoon (or fork)	457 (1.4)	20 (0.4)	437 (1.6)
Missing	6 (0.02)	0 (0)	6 (0.02)
No tooth‐brushing habit	2625 (8.2)	254 (4.6)	2371 (8.9)
Missing	23 (0.07)	1 (0.02)	22 (0.08)
Unable to dress without help	6098 (19.0)	452 (8.2)	5646 (21.2)
Missing	20 (0.06)	3 (0.05)	17 (0.06)
Parental characteristics			
Mean maternal age at delivery, years (SD)^a^	31.7 ± 4.7	31.2 ± 4.6	31.9 ± 4.7
Mean paternal age at delivery, years (SD)^a^	33.6 ± 5.6	33.1 ± 5.6	33.7 ± 5.5
Maternal educational level, *n* (%)^b^			
University or higher	8257 (25.7)	1490 (27.0)	6767 (25.4)
Junior college or vocational school	12 582 (39.1)	2137 (38.7)	10 445 (39.2)
High school	8072 (25.1)	1325 (24.0)	6747 (25.3)
Junior high school and others	1357 (4.2)	229 (4.2)	1128 (4.2)
Missing	1900 (5.9)	336 (6.1)	1564 (5.9)
Paternal educational level, *n* (%)^b^			
University or higher	13 432 (41.8)	2220 (40.3)	11 212 (42.1)
Junior college or vocational school	5489 (17.1)	973 (17.6)	4516 (16.9)
High school	8976 (27.9)	1524 (27.6)	7452 (28.0)
Junior high school and others	1909 (5.9)	352 (6.4)	1557 (5.8)
Missing	2362 (7.3)	448 (8.1)	1914 (7.2)
Residential region, *n* (%)^a^			
Wards	9277 (28.8)	1576 (28.6)	7701 (28.9)
Cities	20 325 (63.2)	3458 (62.7)	16 867 (63.3)
Town or village	2566 (8.0)	483 (8.8)	2083 (7.8)

Abbreviation: SD, standard deviation.

^a^ Obtained from birth records.

^b^ Obtained from the second survey (at the age of 1.5 years).

^c^ Obtained from the third survey (at the age of 2.5 years).

Compared to the diaper‐free group, the diaper‐using group had a higher proportion of boys (53.0% vs. 44.1%) and a lower proportion of children who were second or later in birth order (52.1% vs. 57.9%). The diaper‐using group also had a higher prevalence of low birth weights (9.7% vs. 7.1%) and preterm births (5.5% vs. 3.6%).

In terms of behavioral development, daycare attendance was less common in the diaper‐using group than in the diaper‐free group (33.2% vs. 49.9%, respectively). Additionally, developmental delays were more frequent among children in the diaper‐using group than in the diaper‐free group across all domains: gross motor skills (“unable to climb stairs independently,” 2.3% vs. 0.7%, respectively), language development (“unable to speak in two‐word phrases,” 5.1% vs. 0.8%, respectively), and social habits (“unable to dress or undress independently,” 21.2% vs. 8.2%, respectively).

### 3.2 Selection Bias Assessment

The demographic characteristics of the participants in the final analysis (*n* = 27 220; participants with complete data for all variables in Model 2) and the children lost to follow‐up (*n* = 4948) are presented in Table 2. Among birth‐related variables, the difference between the sexes was significant, with boys being slightly more common in the sample of participants in the final analysis. No substantial differences were found between the other birth‐related variables.

**TABLE 2:** Demographic characteristics of the participants in the final analysis and children lost to follow‐up.

	Final analysis sample (*n* = 27 220)	Lost to follow‐up (*n* = 4948)	*p*
Birth information			
Sex, *n* (%)			
Boys	14 081 (51.7)	2481 (50.1)	0.04*
Girls	13 139 (48.3)	2467 (49.9)	
Birth order, *n* (%)^a^			
First	12 774 (46.9)	2302 (46.5)	0.6
Second or later	14 446 (53.1)	2646 (53.5)	
Birth weight, *n* (%)^a^			
< 2500 g	2499 (9.2)	484 (9.8)	0.18
≥ 2500 g	24 721 (90.8)	4464 (90.2)	
Gestational age, *n* (%)^a^			
Term (≥ 37 weeks)	25 820 (94.9)	4672 (94.4)	0.21
Preterm (< 37 weeks)	1400 (5.1)	276 (5.6)	
Behavioral development and other factors at 2.5 years of age			
Daycare attendance, *n* (%)^c^			
No	17 485 (64.3)	3065 (62.0)	< 0.01*
Yes	9724 (35.7)	1880 (38.0)	
Gross motor skills^c^			
Unable to walk alone	85 (0.3)	30 (0.6)	< 0.01*
Unable to run	157 (0.6)	53 (1.1)	< 0.01*
Unable to climb stairs without help	527 (1.9)	127 (2.6)	< 0.01*
Language development^c^			
Unable to say meaningful words	185 (0.7)	56 (1.1)	< 0.01*
Unable to speak in two‐word phrases	1163 (4.3)	232 (4.7)	0.19
Unable to say own name	3120 (11.5)	547 (11.1)	0.41
Social skills and lifestyle habits^c^			
Unable to eat without help using a spoon (or fork)	378 (1.4)	79 (1.6)	0.26
No tooth‐brushing habit	2144 (7.9)	481 (9.7)	< 0.01*
Unable to dress without help	5226 (19.2)	872 (17.6)	< 0.01*
Parental characteristics			
Mean maternal age at delivery, years (SD)^a^	32.0 ± 4.6	30.7 ± 5.1	< 0.01*
Mean paternal age at delivery, years (SD)^a^	33.8 ± 5.5	32.7 ± 5.9	< 0.01*
Maternal educational level, *n* (%)^b^			
University or higher	7481 (28.6)	776 (19.1)	< 0.01*
Junior college or vocational school	10 991 (41.9)	1591 (39.2)	
High school	6711 (25.6)	1361 (33.5)	
Junior high school and others	1023 (3.9)	334 (8.2)	
Paternal educational level, *n* (%)^b^			
University or higher	12 037 (46.6)	1395 (35.2)	< 0.01*
Junior college or vocational school	4743 (18.4)	746 (18.8)	
High school	7560 (29.2)	1416 (35.8)	
Junior high school and others	1507 (5.8)	402 (10.2)	
Residential area, *n* (%)^a^			
Wards	7912 (29.1)	1365 (27.6)	0.053
Cities	17 164 (63.0)	3161 (63.9)	
Town or village	2144 (7.9)	422 (8.5)	

Abbreviation: SD, standard deviation.

^a^ Obtained from birth records.

^b^ Obtained from the second survey (at the age of 1.5 years).

^c^ Obtained from the third survey (at the age of 2.5 years).

* *p* < 0.05.

For developmental indicators, developmental delays were generally more common among children lost to follow‐up, with mixed patterns across domains. Significant delays were observed in all gross motor indicators in the lost‐to‐follow‐up group, while language and social skills showed variable patterns.

Significant differences were observed in parental characteristics between final analysis participants and children lost to follow‐up. Mean ± standard deviation maternal ages were 32.0 ± 4.6 years vs. 30.7 ± 5.1 years (*p* < 0.01), and paternal ages were 33.8 ± 5.5 years vs. 32.7 ± 5.9 years (*p* < 0.01). The proportion of parents with university education or higher was significantly lower among children lost to follow‐up (*p* < 0.01).

### 3.3 Prevalence of Bedwetting

Table 3 presents the crude and adjusted RRs for the association between bladder control status at 2.5 and bedwetting at 4.5 years of age. Bedwetting prevalence was 42.2% overall (11 497/27 220), 34.5% in diaper‐free children, and 43.9% in diaper‐using children.

**TABLE 3:** Crude and adjusted risk ratios for the association between daytime bladder control status at 2.5 years and bedwetting at 4.5 years.

	Non Bedwettting (*n* = 15 723), *n*/*N* (%)	Bedwettting (*n* = 11 497), *n*/*N* (%)	Crude RR (95% CI)	Model 1 RR (95% CI)	Model 2 RR (95% CI)
Diaper‐Free	3080/4703 (65.5%)	1623/4703 (34.5%)	1 (ref.)	1 (ref.)	1 (ref.)
Diaper‐Using	12 643/22 517 (56.1%)	9874/22 517 (43.9%)	1.27 (1.22–1.33)*	1.24 (1.19–1.30)*	1.25 (1.20–1.31)*

*Note:* Model 1: Adjusted for sex, low birth weight, birth order, preterm birth, parents' age at delivery, parents' educational level, and place of residence at birth. Model 2: Adjusted for sex, low birth weight, birth order, preterm birth, parents' age at delivery, parents' educational level, place of residence at birth, daycare attendance, climbs stairs without assistance, speaks in two‐word phrases, dresses and undresses without assistance.

Abbreviations: CI, confidence interval; ref., reference; RR, risk ratio.

* *p* < 0.001.

### 3.4 Association Between Bladder Control Status and Bedwetting

In the unadjusted analysis, children using diapers at 2.5 years had a significantly higher risk of bedwetting at 4.5 years compared to the diaper‐free group (RR = 1.27, 95% CI: 1.22–1.33, *p* < 0.001).

After adjustment for perinatal factors (Model 1), the association remained significant (RR = 1.24, 95% CI: 1.19–1.30, *p* < 0.001). Further adjustment for postnatal factors (Model 2) yielded similar results (RR = 1.25, 95% CI: 1.20–1.31, *p* < 0.001).

### 3.5 Sensitivity Analysis

A sensitivity analysis was conducted to evaluate the robustness of our findings by treating bedwetting as a three‐level categorical variable (“Never,” “Sometimes,” and “Often”), and using a multinomial logistic regression model. This approach allowed us to examine whether the categorization of bedwetting influenced the observed associations.

The results, presented in Table 4, demonstrate that diaper use at 2.5 years was significantly associated with both “Sometimes” and “Often” bedwetting at 4.5 years compared to “Never” bedwetting. In Model 2, adjusted odds ratios were 1.41 (95% CI: 1.30–1.52) for “Sometimes” and 1.58 (95% CI: 1.42–1.77) for “Often” bedwetting. Similar associations were observed in the crude and Model 1 analyzes, with consistent statistical significance across all models.

**TABLE 4:** Multinomial logistic regression analysis of the association between daytime bladder control status at 2.5 years and bedwetting frequency at 4.5 years (*N* = 27 220).

	“Never” vs. “sometimes” OR (95% CI)	“Never” vs. “often” OR (95% CI)
Crude		
Diaper‐Free	1 (ref.)	1 (ref.)
Diaper‐Using	1.41 (1.31–1.51)*	1.68 (1.51–1.87)*
Model 1		
Diaper‐Free	1 (ref.)	1 (ref.)
Diaper‐Using	1.37 (1.27–1.48)*	1.59 (1.42–1.77)*
Model 2		
Diaper‐Free	1 (ref.)	1 (ref.)
Diaper‐Using	1.41 (1.30–1.52)*	1.58 (1.42–1.77)*

*Note:* Model 1: Adjusted for sex, low birth weight, birth order, preterm birth, parents' age at delivery, parents' educational level, and place of residence at birth. Model 2: Adjusted for sex, low birth weight, birth order, preterm birth, parents' age at delivery, parents' educational level, place of residence at birth, daycare attendance, climbs stairs without assistance, can speak in two‐word phrases, and dresses and undresses without assistance.

Abbreviations: CI, confidence interval; OR, odds ratio.

* *p* < 0.001.

## 4 Discussion

### 4.1 Main Findings

This longitudinal cohort study demonstrated that children who had not achieved daytime bladder control at age 2.5 years showed a 25% increased risk of bedwetting at age 4.5 years, after accounting for developmental milestones and socioeconomic factors. The interpretation of this association requires caution, as continued diaper use at 2.5 years may not solely reflect a marker of bladder control developmental status, but could also represent toilet training practices, a direct effect of diaper use itself, or a combination of these factors. As an observational study, we cannot definitively distinguish between these mechanisms.

The consistency of risk ratios across both crude and adjusted models suggests that the observed association is unlikely to be strongly confounded by included covariates. Also, sensitivity analysis revealed a dose–response relationship, with stepwise increases in risk according to bedwetting severity (mild: aOR 1.41, severe: aOR 1.58), demonstrating that this association is not merely binary but exhibits a graded relationship.

The longitudinal design of this study established a temporal sequence, with exposure assessment at 2.5 years preceding outcome evaluation at 4.5 years, thus eliminating the possibility of reverse causation that plagues cross‐sectional studies. These strengths support the validity of our findings.

### 4.2 Comparison With Previous Studies

Our findings are consistent with previous observational studies reporting associations between daytime continence and subsequent NE. Kawauchi et al. [10] longitudinally demonstrated that daytime urinary incontinence at age 3 years predicts NE at age 5 years, which aligns with our findings. Also, multiple reviews and guidelines [6, 16] acknowledge the developmental relationship between daytime and nighttime bladder control.

However, causality remains unclear. A systematic review by de Carvalho Mrad et al. [17] concluded that there is no clear evidence that any specific toilet training method or timing is superior for preventing NE. Similarly, while observational studies from Asian populations where early toilet training is culturally normative report lower NE prevalence [18], this may reflect complex cultural and social factors rather than intervention effects.

Therefore, bladder control status at 2.5 years may have value as a “marker” for predicting subsequent bedwetting risk, but its validity as an “intervention target” has not been established.

### 4.3 Biological Mechanisms

In bladder control development, the period from 2.5 to 4.5 years is critically important. Jansson et al. [7] demonstrated that bladder capacity increases substantially during early childhood, with the most obvious increase occurring between ages 2 and 3 years, underscoring the importance of this period as critical for sensory perception and voluntary control acquisition. Also, Touchette et al. [13] reported that most improvements in NE occur between 29 and 41 months. These findings support that the period from 2.5 to 4.5 years that we focused on represents a sensitive period for bladder function development.

Prolonged diaper use during this sensitive period may reduce exposure to wetness cues. Neuroimaging studies reported that children with NE show reduced functional connectivity in bladder‐related brain regions (e.g., prefrontal cortex, thalamus) [19]. Based on these findings, some researchers have suggested that reduced sensory input from diaper use may affect bladder‐brain connectivity development, potentially increasing bedwetting risk [20]. However, this mechanism remains unproven with unclear causality.

Our research demonstrated the association between daytime bladder control status during this critical developmental period and subsequent nighttime bladder function. This provides a biological context for the observed association between diaper‐free status at 2.5 years and the prevalence of NE at 4.5 years.

### 4.4 Cultural and Environmental Factors

In addition to these biological mechanisms, environmental and cultural factors may play important roles in bladder control development. Cultural practices affect NE prevalence and toilet training approaches, varying markedly between regions. NE prevalence is lower in Asia, where early toilet training is more common [18]. Many Asian countries use established behavioral practices incorporating early toilet training [21], while Western countries often follow child‐oriented approaches, waiting for readiness before initiating training [17]. The high proportion still using diapers at 2.5 years (82.8%) in our cohort may reflect shifting cultural practices toward child‐readiness approaches in modern Japan.

In Japan, the high proportion of daycare attendance among children aged 1–2 years (60.9%) [22] and group‐based routines may contribute to an earlier establishment of elimination rhythms. Our study found that children in the diaper‐free group were more likely to attend daycare, which suggests a link between group environments and urinary independence. This potential association warrants further investigation.

### 4.5 Clinical Implications

Our findings suggest that children still using diapers at 2.5 years may benefit from developmental assessment rather than intensive toilet training interventions. The association with later bedwetting should be interpreted as a potential marker for identifying children who may need additional support, not as evidence that forced early toilet training prevents bedwetting. Health examinations could incorporate urinary habit evaluations.

While our study shows an association between continued diaper use at 2.5 years and bedwetting at 4.5 years, this does not mean that early diaper removal will prevent bedwetting. Parents concerned about their child's toileting development should consult healthcare providers for individualized guidance.

### 4.6 Study Limitations

This study has several limitations. First, selection bias may have occurred as children lost to follow‐up (*n* = 4948) had different demographic and developmental characteristics compared to those in the final analysis. Children lost to follow‐up had more developmental delays and lower parental education—known bedwetting risk factors [23]—though bias direction remains unclear. This limits generalizability. Second, differential reporting bias may have occurred, as parents of children with prolonged daytime diaper use might demonstrate heightened awareness of nighttime voiding issues, potentially overestimating the association. Third, information bias may have occurred because both exposure and outcome variables relied on parental self‐reporting. Fourth, we cannot rule out unmeasured confounding factors, such as genetic predisposition or family history of bedwetting. Fifth, we cannot distinguish between children whose continued diaper use at 2.5 years reflects developmental delays and those whose parents have not yet initiated toilet training. This distinction affects interpretation as prognoses may differ. Sixth, the assessment at 4.5 years, while capturing an important developmental period, precedes the formal diagnostic age for NE (5 years). Some may achieve spontaneous resolution before NE diagnosis age. Finally, the findings may have limited generalizability to populations with different cultural practices regarding toilet training or healthcare systems.

This study, using a nationally representative sample of over 30 000 children and standardized survey data, demonstrated an association between daytime bladder control status at 2.5 years and bedwetting risk at 4.5 years with high statistical power and measurement consistency. This association likely reflects individual differences in bladder control maturation, and the observed dose–response relationship supports the robustness of our findings. Future research should clarify the mechanisms underlying this developmental relationship and establish optimal support strategies tailored to individual children's developmental stages.

## Author Contributions

**Takatoshi Moriwake:** conceptualization, writing – review and editing, writing – original draft. **Naomi Matsumoto:** conceptualization, data curation, writing – review and editing, formal analysis. **Yusuke Tominaga:** writing – review and editing. **Kensuke Uraguchi:** conceptualization, data curation, writing – review and editing. **Tomoko Kobayashi:** writing – review and editing. **Ichiro Tsuboi:** investigation. **Kasumi Yoshinaga:** investigation. **Tomoaki Yamanoi:** investigation, writing – review and editing. **Tatsushi Kawada:** investigation. **Takuya Sadahira:** investigation, writing – review and editing. **Satoshi Katayama:** investigation, writing – review and editing. **Takehiro Iwata:** investigation. **Shingo Nishimura:** investigation. **Kensuke Bekku:** investigation. **Kohei Edamura:** investigation. **Soshi Takao:** formal analysis, methodology. **Takashi Yorifuji:** methodology, formal analysis, supervision. **Motoo Araki:** supervision.

## Ethics Statement

This study was approved by the Institutional Review Board of Okayama University Hospital (Approval No. 1506–073).

## Consent

The authors have nothing to report.

## Conflicts of Interest

Motoo Araki is an Editorial Board member of the International Journal of Urology and a co‐author of this article. To minimize bias, he was excluded from all editorial decision‐making related to the acceptance of this article for publication. The other authors declare no conflicts of interest.
